# Advanced mid-infrared lightsources above and beyond lasers and their analytical utility

**DOI:** 10.1007/s44211-022-00133-3

**Published:** 2022-07-03

**Authors:** Michael Hlavatsch, Boris Mizaikoff

**Affiliations:** 1 Institute of Analytical and Bioanalytical Chemistry, Ulm University, Albert-Einstein-Allee 11, 89081 Ulm, Germany; 2 Hahn-Schickard, Institute for Microanalysis Systems, Sedanstrasse 14, 89077 Ulm, Germany

**Keywords:** Mid-infrared, Thermal emitter, MIR, Light emitting diode, MIR-LED, Infrared sensors, Infrared diagnostics

## Abstract

In the mid-infrared (MIR) spectral range, a series of applications have successfully been shown in the fields of sensing, security and defense, energy conservation, and communications. In particular, rapid and recent developments in MIR light sources have significantly increased the interest in developing MIR optical systems, sensors, and diagnostics especially for chem/bio detection schemes and molecular analytical application scenarios. In addition to the advancements in optoelectronic light sources, and especially quantum and interband cascade lasers (QCLs, ICLs) largely driving the increasing interest in the MIR regime, also thermal emitters and light emitting diodes (LEDs) offer opportunities to alternatively fill current gaps in spectral coverage specifically with analytical applications and chem/bio sensing/diagnostics in the focus. As MIR laser technology has been broadly covered in a variety of articles, the present review aims at summarizing recent developments in MIR non-laser light sources highlighting their analytical utility in the MIR wavelength range.

**Graphical abstract:**

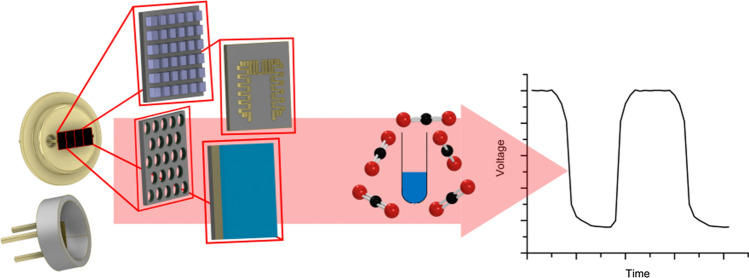

## Introduction

The infrared (IR) spectral regime provides particularly pronounced molecular information due to the excitation of molecular vibrational, ro-vibrational, and rotational transitions. Generally, the MIR range is defined as the regime of electromagnetic (EM) waves with a wavelength extending from approx. 2 to 20 µm. Adjacent, the near-infrared (NIR) and the far-infrared (FIR) are extending towards the shorter wavelength (approx. 800 nm to 2 µm) and the longer wavelength (approx. 20 µm to 3 mm) regime, respectively. In the analytical MIR, it is common practice that wavenumber units (i.e., inverse wavelength) are used instead of the wavelength for direct scaling with the photon energy. Across disciplines (e.g., physics, electrical engineering, etc.) and in literature, the MIR is frequently further divided into the short-wave infrared (SWIR, 1.4–3 µm), the mid-wave infrared (MWIR, 3–8 µm), and the long-wave infrared (LWIR, 8–20 µm), respectively. When illuminating samples with MIR radiation, fundamental rotational and vibrational resonances within virtually any kind of molecule—with few exceptions—independent of molecular weight, complexity, dimension, physical status (i.e., gas, liquid, semi-solid, solid) or nature (i.e., organic and inorganic) are excited. Hence, the MIR spectrum of a sample is frequently referred to as the ‘molecular fingerprint’ enabling the detection and quantification of a wide range of relevant molecular compounds in environmental, health and medical, industrial, and security and defense applications [1–3].

Historically, most MIR studies have been performed using Fourier-transform infrared (FTIR) spectrometers, which use broadband thermal light sources and an interferometer for radiation modulation generating high-resolution reflectance, transmission or absorption spectra. Broadband MIR radiation is typically provided by a heated material (e.g., SiC filament) covering the entire MIR spectral range. However, due to the nature of broadband blackbody radiators, the energy density per wavelength remains limited. Additionally, such radiation sources are inefficient (i.e., in terms of optical output) and not compatible with applications that require either low power consumption or highly directional, narrowband, or coherent MIR radiation.

Nowadays, specific molecular transitions in the MIR regime may advantageously be excited via narrowband light sources such as quantum cascade—or interband cascade—lasers (QCLs, ICLs).

QCLs were first experimentally shown in 1994 [4], and are based on intersubband transitions between engineered conduction band states in complex semiconductor heterostructures instead of conventional electron–hole recombination within a semiconductor bandgap [5, 6]. These electronic transitions lead to efficient photon emission in the MIR range and enable a variety of spectroscopic target applications [7, 8]. However, QCLs are limited to wavelengths λ > 5 μm due to the common use of the InGaAs/InAlAs material system.

An alternative to QCLs are so-called interband cascade lasers (ICLs), which were proposed and realized around the same time as QCLs [9, 10]. ICLs are based on electron–hole recombination of type-ΙΙ interfaces, whereby as an example electrons of the InAs conduction band recombine with holes of the InGaSb valence band. With this design, it is possible to operate ICLs in continuous-wave (cw) mode at room temperature with low electrical power requirements (vs. QCLs) within a spectral window of 3–7 µm. However, these laser sources are complex and expensive to develop, and are therefore of limited utility for low-cost system development. Power-hungry QCLs likewise have limitations for miniaturized and/or low-cost systems.

In this review, we discuss latest MIR light sources above and beyond these nowadays established and proven laser systems, and provide an overview on alternative MIR emitters and their potential analytical utility in future low-cost, low-power, narrow-band MIR sensing and diagnostic technologies. It should be noted that indeed this review is of rather technical nature, however, it is important to summarize, compare, and understand advances of these emitters vs. laser technology for highlighting their importance in molecular analytical applications.

## Advancements in MIR light sources beyond lasers

### Thermal emitters

Thermal emitters are structures that emit electromagnetic radiation in the MIR range by heating to approx. 200–1400 K. The emitted surface radiation can be described via Planck’s law:

1 $${\text{E}}\left( {\lambda ,\,{\text{T}}} \right) = \; \in \left( {\lambda ,\,{\text{T}}} \right)\frac{{2\pi {\text{hc}}^{2} }}{{\lambda^{5} }}\frac{1}{{{\text{e}}^{{{\text{hc/}}\lambda {\text{kT}}}} - 1}},$$

where *λ* is the wavelength of the emitted light, *T* the temperature of the object, *h* Planck’s constant, *c* the speed of light, *k* the Boltzmann constant and ϵ (λ, T) the emissivity.

The emissivity ϵ (λ, T) describes the spectral emittance of a blackbody as a function of wavelength and temperature, whereby the limit ϵ (λ, T) = 1 represents the case of an ideal blackbody radiator. Typical blackbody emitters are the Nernst glower and the globar, whereby either a ceramic rod (e.g., SiC) or a tungsten filament, respectively, are resistively heated.

**Fig. 1:**
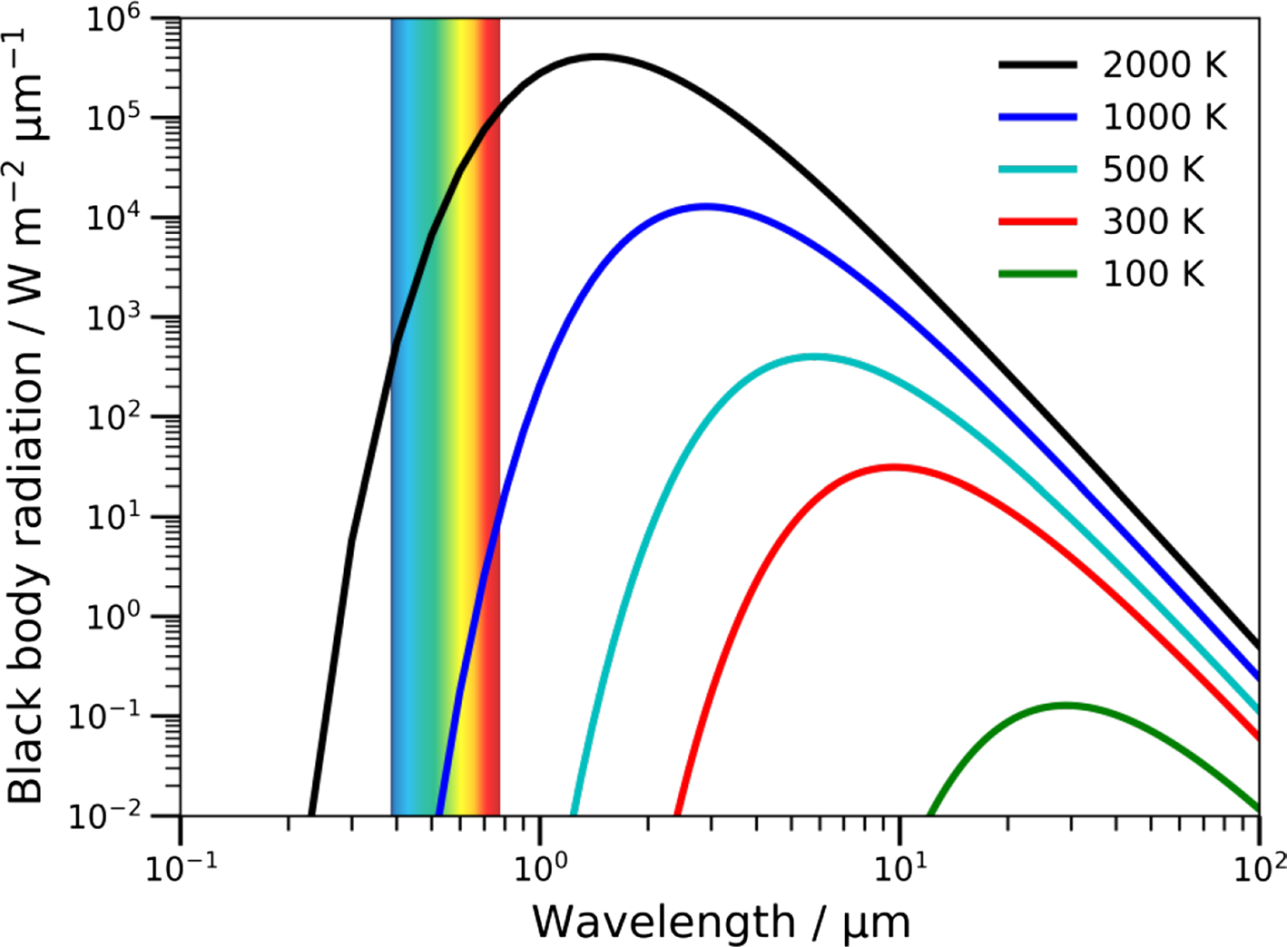
Blackbody radiation for various temperatures in the range 100–2000 K

Even though thermal emitters are inexpensive, the emitted radiation—central wavelength and radiant intensity—can only be controlled by the temperature of the emitter element. Figure 1 shows the blackbody radiation of an ideal emitter and the temperature dependence of the emitted radiation in terms of wavelength. This dependence renders thermal emitters for specific applications in the fingerprint region (e.g., 6–15 µm) of limited utility, as for this spectral regime a thermal emitter would have to be operated at room temperature, and thus would have only low emissivity. To overcome these limits, one may refer to Kirchhoff’s law of radiation. Kirchhoff's law states that the emissivity of a blackbody radiator at a given temperature and wavelength is equal to the ratio of emissivity ϵ and absorptivity *α* at that same wavelength and temperature constant:

2 $$\in \left( {\lambda ,\,{\text{T}},\,{\uptheta }} \right) = {\upalpha }\left( {\lambda ,\,{\text{T}},\,{\uptheta }} \right) = 1 - {\text{R}} - {\text{T}},$$

where *R* is the reflectivity, *T* the transmissivity and *θ* the angle of incidence of the light. Thus, following from Kirchhoff's law an adequate absorber must also be an adequate blackbody emitter, which can be exploited for the development of advanced thermal emitters. Despite evident progress in light source technology, a large number of the systems reported to date are based on either a change of a specific property of a natural material or a temperature change, and are, therefore, limited by operation at high temperatures, a limited operating frequency, or a slow modulation speed.

In recent years, advances in nanofabrication and coating techniques have led to the development of deliberately design and tailorable surface structures (i.e., especially so-called metamaterials), which promise approaches for also improving MIR thermal radiation emission. By applying metamaterial structures, selective spectral losses are generated for controlling the emissivity, and consequently, the heat of the thermal emitter.

Customizing the dielectric constant and permeability of metamaterials by deliberately tailoring the geometry, dimension, and material composition along with periodic patterns leads to novel optical properties enabling the control of electromagnetic waves at the sub-wavelength level.

In the following, we will discuss in more detail the development of metamaterial-based thermal emitters and their potential applications.

#### Microelectromechanical system (MEMS)-based thermal emitters

MEMS were first introduced in the mid-1980s [11]. MEMS devices are usually structured from components at the 1–100 µm scale, and the dimensions of the entire resulting device is in the range of few micrometers up to several millimeters [12]. These components are usually fabricated from materials including polymers, metals, silicon—and other semiconductors—and ceramics, and are structured and fabricated using various lithographic, etching, and deposition methods [13–15].

Shortly after their fundamental introduction, the first MEMS thermal emitters for sensor applications were reported [16–18]. Parameswaran et al*.* [16] first reported the fabrication of polysilicon microbridges, sandwiched oxide microbridges, and sandwiched oxide cantilever beams using a twin-tub complementary metal–oxide–semiconductor (CMOS), and discussed their potential for sensor applications. In the following, Greenwald et al*.* [19] showed etching periodic structures onto a silicon surface coated with gold by varying the interstitial proportions and geometric patterns. Thereby, the central emission wavelength was controlled and narrowband heat emitters with selected wavelengths were produced. Further research has resulted in the use of advanced structuring techniques and novel materials for the fabrication of MEMS systems. For example, silicon-on-insulator (SOI) wafers (e.g., made of SiO_2_, AnSiO_2_, etc.) were used in MEMS fabrication, and in addition, silicon-based surfaces were no longer coated with metals but also doped with semiconductors (e.g., SnO_2_:Sb, poly-Si, PtSi, Boron-doped poly-Si, etc.) to ensure resistive heating and tailor the properties [13–15, 20–23].

**Fig. 2:**
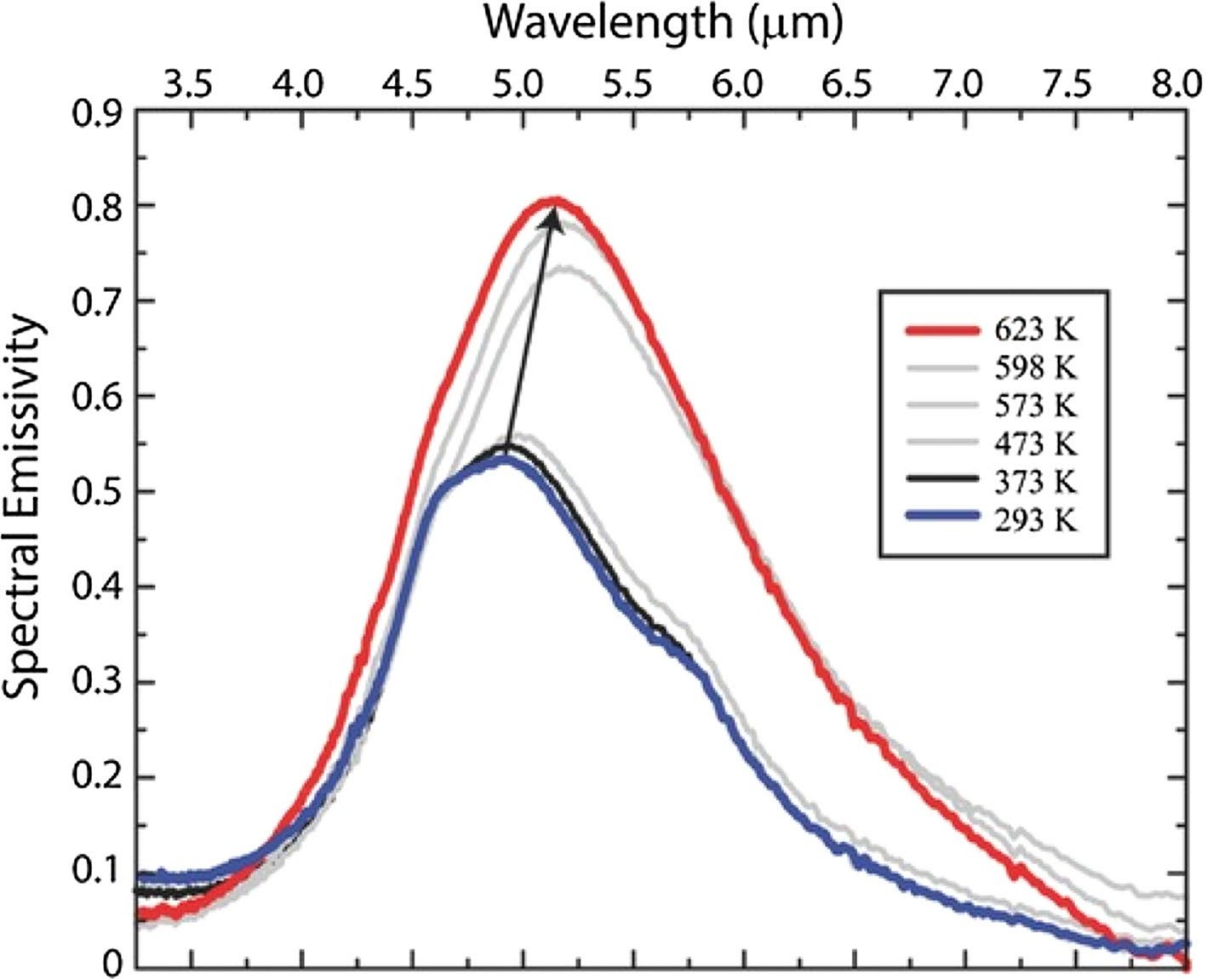
Emitted power density of the thermochromic metamaterial. Power density per wave-length for different temperatures as indicated in the inset legend. Copyright © 2015, John Wiley and Sons. Reprinted and edited with permission from [28]

Most recent research has focused on increasing the emissivity at high temperatures (> 300 °C) by tuning the MEMS structure using gold metamaterials on SiO_2_ [24, 25]. Again, it was shown that by varying the structure thickness and pattern spacing, the central emission wavelength and the intensity of the thermal radiation changes depending on these parameters. Furthermore, it has been shown by that by depositing graphene oxide (GO) using the CMOS process, the intensity of the emitted radiation may be further enhanced [26, 27]. Chen and Chen [26] as well as Li et al*.* [27] showed that by applying GO coatings at the thermal emitter surface, an increase of up to 150% of the signal can be achieved. Furthermore, Liu and Padilla demonstrated two distinctly different MEMS emitters with various geometric designs and central emission wavelengths (~ 5 and ~ 8.5 µm, respectively), which have spectral radiant energy densities of up to 2.0∙10^7^ W/m^3^ at room temperature [28, 29]. Fig. 2 shows the temperature dependence and wavelength shift of spectral emissivity as a function of applied temperature for the above MEMS emitter.

Despite the significantly lower radiant energy density compared to thermal emitters operated at much higher temperatures, these advancements demonstrate the potential of novel metamaterial-based MEMS emitters at room temperature operation, which renders them ideally suited for integration into miniaturized sensing systems and diagnostics.

#### Plasmonic nanostructured arrays

Plasmonic nanostructured arrays take advantage of the fact that the electrons in these structures that want to restore the ground state are prevented from moving by the oscillation of the EM wave, and are instead stimulated to oscillate at the frequency of the EM radiation resulting in a shift of the dipole.

In 1998, Ebbesen and colleagues have for the first time been able to show transmission in the NIR resulting from perforated metallic films via plasmonic structures [30, 31]. For this purpose, an Ag film of thickness 0.2 µm or between 0.2 and 0.5 µm, respectively, was deposited onto a quartz surface by evaporation, and subsequently arrays of cylindrical holes were fabricated via focused ion beam (FIB) milling. Later, first plasmonic thermal emitters in the mid-infrared regime were also published. In contrast to Ebbesen and colleagues, here a metal/insulator/metal layer (e.g., Ag/SiO_2_/Ag, Au/SiO_2_/Au, nanoamorphous carbon:TiN/SiO_2_/nanoamorphous carbon:TiN) [32–38] or a graphene/insulator layer (e.g., graphene/SiO_2_, graphene/diamond-like carbon, SiN_x_) [39, 40] was deposited onto a silicon wafer and arrays of periodic nanostructures (e.g., cylinders, hexagons, gratings, ribbons) were fabricated.

These plasmonic emitters have a lattice constant of 1.5–5 µm for non-graphene structures, and a lattice constant of 40–200 nm for graphene structures in common. Furthermore, the respective metal/insulator/metal (MIM) layers vary between 20 and 300 nm with the insulator layer predominantly in the range of 20–100 nm. As with MEMS-based emitters, the variation of the structural parameters (i.e., lattice constant, layer thickness, structure, material, etc.) selective thermal emitters may be tailored whose intensity increases with increasing surface temperature. Beyond that, Si-Chen Lee and co-workers [41, 42] have developed a plasmonic thermal emitter with dual and triple wavelength emission, respectively. For their dual-wavelength emitter, they fabricated a double triple-layer stacked Au/SiO_2_/Au/SiO_2_/Au structure, which resulted in emission maxima at 4.75 µm and at 6.45 µm by coupling the SiO_2_ layer-generated blackbody radiation with the modes of localized surface plasmons (LSP). In turn, the plasmonic thermal emitter with triple wavelength emission was obtained by a periodic arrangement of SiO_2_ and embedding TiO_2_ strips in SiO_2_. They have shown that this arrangement leads to LSP modes with multiple orders, and the LSP emission wavelength can be controlled by adjusting the thickness and linewidth of the TiO_2_ strips.

More recently, polymers have been introduced as materials for the fabrication of plasmonic thermal emitters. Moridani et al*.* [43] fabricated emitters based on structured formation the of metallic layers at elastomeric substrates. For this purpose, they deposited a nickel layer onto a 1.5 mm thick, cured and uniaxially stretched polydimethylsiloxane (PDMS) substrate. Due to the buckling instability of PDMS, regularly recurring wrinkle patterns spontaneously formed in the direction perpendicular of the stretching direction once the PDMS substrate was relaxed to its initial dimensions. The periodicity of the wrinkle pattern was designed by controlling the thickness of the deposited nickel layer and fabricating three different periodicities of 4.5, 6.3, and 9.4 µm. Finally, to achieve emissivity in the MIR region, they coated the nickel-coated structure with a 50 nm thick gold layer after the wrinkles were formed. Their measurements showed that the plasmonic thermal emission of the respective periodicities emitted infrared radiation with maxima at 4.5, 6.3 and 9.4 µm depending on the periodicity itself and the orientation. Lee et al. [44], have proposed a hybrid thermal emitter (HTE) based on spoof surface plasmons of microscopic Ag grooves manifested in encapsulated polymer layers. To that end, they etched a groove structure with a periodicity of 10 µm and a depth of 1.7 µm into a Si-wafer using photolithography and an inductively coupled plasma spectrometer. This structure was then vapor-deposited with a 100 nm thick Ag layer, and subsequently, a 3 µm thick encapsulation layer consisting of styrene–ethylene–butylene–styrene (SEBS) was spin-coated onto the metal layer. Their simulated and measured emission spectra showed that on the one hand a thin SEBS film is indeed IR-transparent, and on the other hand that the Ag lattice array is poorly visible in the IR. However, when combined as an HTE enhanced spatially resolved emission was evident due to the entangled spoof surface plasmons.

#### Thin film-based thermal emitters

Thin film technology found its way into many applications such as waveguides [7, 8], interlayers to achieve stronger adhesion in metallic films [45, 46], and as protective coatings [47]. A type of thin film-based thermal emitter is based on an aperiodic multilayer structure. This concept comprises a thin structure consisting of a non-periodic number and thickness of alternating layers of a conductive material and an insulator. For example, Campione et al. [48] have developed an aperiodic multilayer emitter consisting of 50 pairs of 10 nm thick doped In_0.53_Ga_0.47_As and 8 nm thick undoped Al_0.48_In_0.52_As layers on an InP substrate with a 200 nm thick Al_0.48_In_0.52_As buffer layer between the substrate and multilayer. Doped In_0.53_Ga_0.47_As and undoped Al_0.48_In_0.52_As behave like metal and non-conductor, respectively. It was found that the generated thermal radiation is in the close to the epsilon-near-zero (ENZ) frequency of the doped quantum wells (QW) of the hyperbolic multilayer. As a result, they showed that the p-polarization of the thermal radiation, which is close to the ENZ frequency (central wavelength at ~ 6.25 µm) has stronger emissivity than for the s-polarization (central wavelength at ~ 9 µm). In contrast, Sharifi et al*.* [49] proposed an aperiodic structure consisting of a graphene/hexagonal-boron-nitride (hBN; insulator) multilayer sandwiched between thick SiC layers on a tungsten substrate. By applying a DC electric field perpendicular to the graphene/hBN surfaces, they have varied the chemical potential in the range of 0–1 eV of graphene and thus optimized the selectivity, tunability and switching ability of the thermal radiation of the multilayer structure for 8, 13, 23, 28 and 32 graphene layers for a central wavelength of ~ 3.34 µm. It was demonstrated that increasing the number of graphene layers enhances the effect of the chemical potential resulting in enhanced tunability. Moreover, by increasing the number of graphene layers, they saw the switching ability increases by changing the chemical potential resulting in a more substantial decrease in thermal output with increasing graphene layers.

**Fig. 3:**
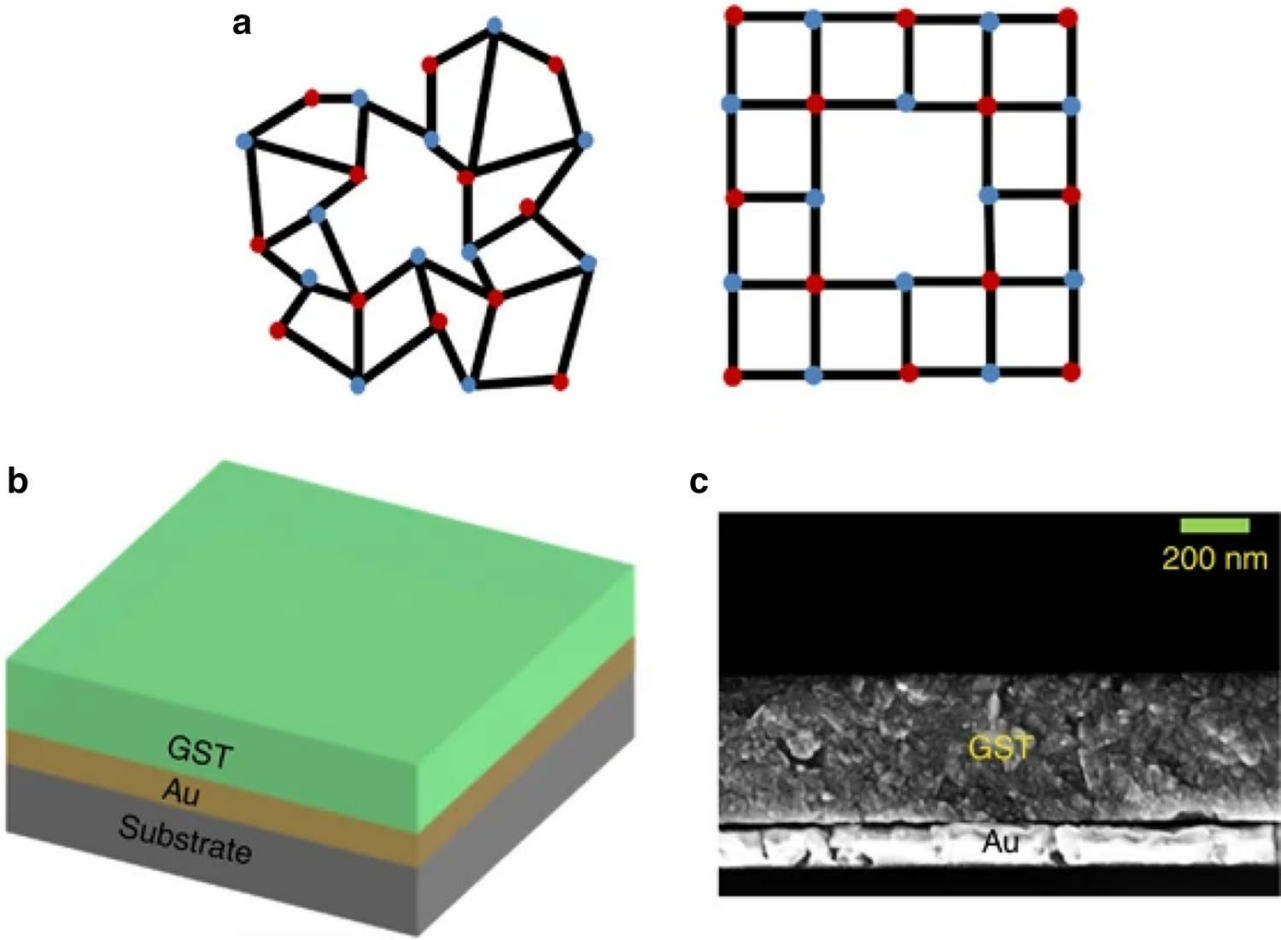
**a** Atom distribution diagrams of the two phases (amorphous and crystalline) of the GST. The red and blue dots denote the Ge/Sb atoms and Te atoms, respectively. **b** A 3D schematic of the switchable and tunable thermal emitter composed of a GST film on top of a gold film. **c** An SEM image of a cross-section of the fabricated thermal emitter. Copyright © 2016, Springer Nature. Reprinted with permission from [53].

In another type of thin-film thermal emitters, the phase-changing property of metamaterials has been utilized to produce heat emitters whose emission can be controlled based on their (dis)ordered molecular structure. For this emitter type, a distinction is made between metallic-dielectric phase transitions and crystalline–amorphous phase transitions. Streyer et al. [50] showed in their system, where a thin film Ge (range 80–600 nm) deposited on a SOI wafer with a 2.5 µm heavily doped Si layer and a 0.9–1.1 µm thick SiO_2_ layer, that by varying the Ge film thickness emission power and the central wavelength can be controlled. Kats et al*.* [51], on the other hand, developed a VO_2_/sapphire system. By exploiting the phase transition of VO_2_ from non-conductive to metallic at approximal 67 °C, thermal radiation could be produced whose peak emissivity is controlled by temperature variation without changing the central wavelength (11.5 µm). Among the crystalline-amorphous phase transition based thermal emitters, GeSb_2_Te_5_ (GST) is of particular interest [52–54]. Although the atoms of GST are chaotically arranged in the amorphous phase (see Fig. 3a), they are arranged in an ordered structure in the crystalline phase resulting in different infrared properties. Du and colleagues [53, 54] have demonstrated in two of their studies the realization and wavelength tunability for different GST based thermal emitters and the ability to maintain high emissivity while performing continuous wavelength tuning. In their first study [53], they showed how varying the GST film on a 120 nm thick Au layer again deposited on substrate the emissivity was switchable and tunable, and how the wavelength selectivity of GST was manipulated. Figure 3b shows a 3D schematic and Fig. 3c shows an SEM image of their fabricated emitters with GST deposited on the thin Au layer. Emissivity control was demonstrated by steadily increasing the temperature from 100 to 170 °C for a crystalline GST thickness of 450 nm. The temperature increase was accompanied by a shift of the central wavelength from ~ 8 to ~ 10.5 µm, which agrees with Wien's law, and reveals an associated increase of the emissivity. Furthermore, they showed the shift of the central wavelength to higher wavelengths (from ~ 8 to ~ 14 µm) by increasing the GST thickness by measuring the emissivity of 360, 450, and 540 nm thick crystalline GST films at 100 °C. To investigate this further, in a second study [54]. Du and colleagues investigated a bilayer emitter consisting of GST-Al and a trilayer emitter of Cr-GST-Au for continuous wavelength matching at consistently high emissivity values (> 0.75 for GST-Al and > 0.63 for Cr-GST-Au emitters, respectively).

The emitters each consisted of a varying GST layer, a 100 nm thick metal layer (Al and Au) and, in the case of the triple layer, a 5 nm thin Cr layer. Again, they were able to control the central wavelength using the GST thickness in the range of 7–13 µm. In addition, they showed that both the duration and temperature of the annealing process affect the emissivity, as it affects the crystallization rate of GST.

#### Photonic crystal-based thermal emitters

In general, photonic crystals (PC) are periodic optical nanostructures that affect the motion of phonons. They can be classified into three-dimensional (3D) [55–59], two-dimensional (2D) [60–64] and one-dimensional (1D) [65–69] PCs. PCs consist of periodic dielectric, metal-dielectric or nanostructures that influence the propagation of electromagnetic waves by defining allowed and forbidden electronic energy bands. The structure of the PC consists of regularly repeating regions of high and low dielectric constant. Wavelengths propagating in the PC structure are called modes, while forbidden wavelengths are called photonic band gaps.

Various research groups have used the woodpile structure for 3D PC [55–59]. For the fabrication of metallic woodpile structure 3D PC, a mold material (e.g., SiO2 or polymers) was patterned, then metal (e.g., tungsten, nickel) was deposited or filled onto the prefabricated mold, the mold material was removed, and, if necessary, further processed. This can be repeated as often as required to obtain the desired number of layers. Fleming and colleagues [55–57] developed and investigated various tungsten 3D PC thermal emitters. The respective PCs consisted of four layers (rod width: 1.2 µm, lattice constant: 4.2 µm), 6 layers (rod width: 0.85 µm, lattice constant: 2.8 µm) and 8 layers (rod width: 0.5 µm, lattice constant: 1.5 µm) of tungsten, respectively. For all their systems, they observed an increase in emission intensity with increasing drive current. Furthermore, for all 3D PC tungsten emitters, they were able to detect a sharp peak (4-layer: ~ 5 µm; 6-layer: ~ 4 µm; 8-layer: ~ 1.6 µm) as well as a photonic band gap after the sharp peak, both of which shift toward the near-infrared as the number of layers increases. They demonstrated that the 3D photonic bandgap of their samples effectively suppresses the infrared component of thermal emitters for λ ≥ λ_Edge_ (wavelength at the maximum emission intensity edge). At the same time, the enhanced photon–matter interaction near the band edge enhances light emission.

In 2D PC emitters, holes (e.g., circular, triangular, square) are drilled into a substrate that is transparent to the wavelength of radiation that the bandgap is designed to block. In a numerical study by Chan et al*.* [60], they have shown the influence of geometry on the emission characterization by performing calculations for 2D metallic periodic PCs. For their calculations, they assumed a metal plate of varying width and thickness was drilled into a circular hole or depression of varying radii. They also investigated a hybrid structure consisting of metal and a circular dielectric puck on top of the metal. They were able to show that periodicity affects waveguide cut-off, waveguide resonances, diffraction peaks, and surface plasmons. For example, an increase in hole radius leads to a decrease in frequency peaks, which in turn is indicative of waveguide cut-off. Furthermore, they saw how hybrid structures of metal and dielectric led to emission enhancement, whose emission peaks can be shifted arbitrarily by changing the lattice constant and operating temperature. Additionally, they calculated that by combining hybrid structures, even materials with multiple emission peaks can be developed.

Ji et al. [62] proposed such a metal dielectric 2D PC emitter. The active emitter element was a 12 µm thick cantilevered Si_3_N_4_/SiO_2_/Si membrane coated with a very thin Pt-metal film. Holes with square patterns with apertures of the order of the wavelength were periodically made in both the metal and silicon. This resulted, by resonant coupling of the incident radiation from the underlying silicon and the surface plasmons of the perforated metal surface, in high optical transmission efficiency. The measured emission spectrum of the PC emitter showed a narrow peak centered at ~ 5.8 µm and its intensity was 75% stronger compared to an emitter without PC. Furthermore, Ji and colleagues also found that an increasing grating constant is accompanied by a shift in the peak wavelength.

**Fig. 4:**
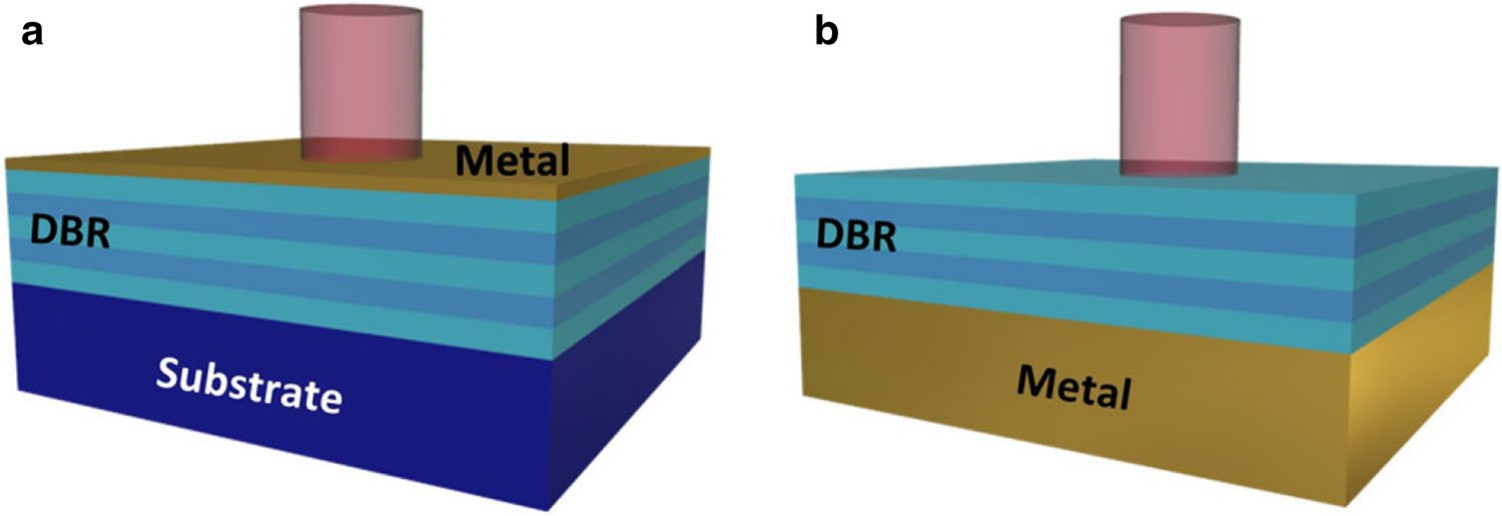
Schematic diagrams of **a** metal-side TPP structure with a thin metal film where light is illuminated from the thin metal layer; **b** DBR-side TPP structure where light is illuminated from the DBR sidewith an opaque metal film below. Copyright © 2017, American Chemical Society. Reprinted with permission from [66].

In terms of PC selective thermal emitter, much research has been done on 1D PC in recent years, as they do not require complex structures and fabrication compared to 2D and 3D PC and have the capability of large area fabrication. In this context, the Tamm plasmons polaritons (TPP) and their optical states are of great interest. These quasi-particles, which can be excited within a distributed Bragg reflector (DBR) and a medium of negative permittivity [70, 71], are capable of improving the properties of thermal emitters [67]. The research group around Yang and colleagues proposed several schemes to realize selective thermal emitters by exploiting 1D PC and the TPP optical states [65, 66, 69]. They proposed various designs to realize selective thermal emitters by exploiting 1D PC and the TPP optical states. For their first proposed thermal emitter [65], they designed a two-pair DBR consisting of Si/SiO_2_ on a Si substrate and a 15 nm thin tungsten film (emitting layer) on the last Si layer of the DBR (outlined in Fig. 4a). The thickness of the Si/SiO2 pair was equal to the quarter optical path length of the target wavelength. By varying the last Si layer (10–150 nm or 10–500 nm) of the DBR, they saw a wavelength shift and, respectively, by changing the tungsten layer (10–50 nm), a broadening of the resonance bandwidth with decreasing metal thickness.

In their subsequent study, Yang et al*.* [66] investigated the ultrasharp emission peak in the MIR of a DBR-sided TPP structure. A DBR-sided structure is sketched in Fig. 4b. DBR-sided TPP structures usually have a thick metal film that behaves like a reflector with strong evanescent fields excited in DBR. In contrast to the metal-sided structure (Fig. 4a), in the DBR-sided one the light incidence is from the DBR and not from the thin metal film. Furthermore, its absorption capacity is controlled by the number of DBR pairs and not by the thickness of the metal film [72]. Yang and coworkers observed a decrease in absorption with increasing DBR pair number for all metals tested. They found the maximum absorption for Al and Au with two DBR pairs, but for Mo and W with one pair. This results from the fact that the coupling efficiency of TPP depends on the optical impedance matching at the interface between DBR and metal. Since Al and Au have higher reflection coefficients than Mo and W, the use of Al or Au supports stronger TPP resonance. In the next step, they investigated the influence of the different metals on the impedance matching. During simulations and experiments, they showed that the plasmonic metals have higher emissivity than the refractory metals. The emissivity of samples with Al was the best, which they could reproducibly prove in a follow-up experiment with different heating voltages.

In their most recent work, they compared a TiN TPP thermal emitter structure (Si/SiO_2_/Si/TiN) with a TiN MIM (TiN/Si/TiN) with a target wavelength of 4 µm. Simulations and experiments showed that the emissivity of the TiN TPP structure is stronger and a higher threshold temperature in vacuum compared to a TiN MIM structure (TiN TPP structure: 1000 °C; TiN MIM: 400 °C). Thus, they proposed another model for wavelength selective thermal emitters with TPP-based structures. Other research groups [67, 68] also showed promising TPP-based structures using Ag as metal and even using semiconductor DBR pairs (ZnSe/Ge), respectively, for 1D photonic crystal structures utilizing the TPP of these metamaterials.

### MIR light emitting diodes (MIR-LEDs)

**Fig. 5:**
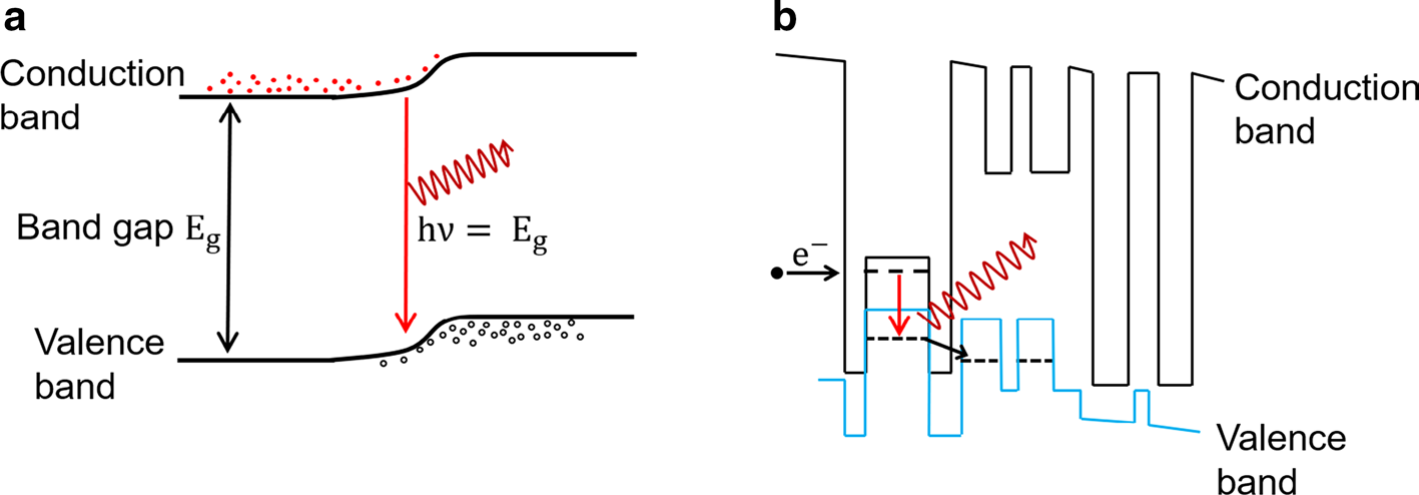
Schematic band structures for **a** conventional light emitting diode, **b** a interband cascade laser ‘W’

The MIR-LEDs are alternative broadband incoherent light sources—albeit significantly narrower than thermal emitters—that offer higher modulation speed and distinct tailorability of the emission spectrum [73–76]. However, the structure of MIR-LEDs is very different from that of conventional LEDs based on electron (${\text{e}}^{ - }$)-hole recombination, as shown in Fig. 5a. Structures suitable for MIR-LEDs present several challenges. A particular challenge is that the use of narrow bandgap materials significantly increases Auger recombination, which results in MIR sources based on narrow bandgap materials being inefficient at the high carrier injection levels required for occupancy inversion at which the most efficient emitters operate. Furthermore, the use of narrow bandgap materials limits the number of alloys available for heterostructure growth. For this reason, light generation in MIR-LEDs is achieved by recombining electrons and holes in heterostructures—similar to ICLs—which typically use a type-II interface (see Fig. 5b). In the “W” element structure, electrons in the conduction band QW recombine with holes in the valence band QWs. As a result, the broken-gap type-II alignment allows emission energies below the bulk band gap of the two components by controlling the QW thickness and thus the position of the electron and hole energy states in the active region where ${\text{e}}^{ - }$–hole recombination occurs.

Type-II interband cascade LED (IC-LEDs) structures in the 5–8 µm spectral region were first reported by Yang et al. [73]. This structure enables circumventing the fast photon relaxation rate in quantum cascade lasers and still maintain cascade tunnel injection and wavelength tunability. To achieve this, they have developed a type-II InAs/GaInSb/AlSb IC structure. The structure was grown by molecular beam epitaxy (MBE) on a GaSb substrate and comprises 15 periods of active regions separated by n-doped injection regions that serve both as collectors for the preceding active regions and as emitters for the subsequent regions. In the following years, the adjustment of the Sb content in the different layers (e.g., InAsSb) led to the extension of the wavelength range. Krier et al*.* [75] achieved ∼4 μm emission at room temperature in their InAs/InAs_0.87_Sb_0.13_ LED system. Around the same time, Haigh et al*.* [76] showed that by changing the aluminum content (here: 0–8.8% Al) in Al_x_In_1-x_Sb LEDs, the wavelength of the peak emission can be adjusted in the wavelength range ~ 3.5–5.5 µm. Later in 2009, Lackner et al. [77]*.* were able to further increase the Sb content in their InAsSb/InAs system to 26.7% Sb and demonstrate their emissivity using 4 K photoluminescence.

Around 2010, several research groups have investigated LED systems based on InAs/Ga(In)Sb. Das [78] and Koerperick et al. [79] showed similar peak wavelengths of ~ 8 µm for an InAs/GaInSb/InAs multi quantum well (MQW) LED and a superlattice (SL) InAs/GaSb LED, respectively. Shortly after the publication mentioned above, Das published another paper [80] showing how varying the indium fraction (18–30%) of the InAs/Ga_1-x_In_x_Sb/InAs structure with decreasing fraction leads to an increase of the emission intensity.

In 2015, Provence et al*.* [81] showed that SL-LEDs grown on mismatched GaAs substrate exhibited higher radiance than those grown on lattice GaSb substrates at a measurement temperature of 77 K. The higher emission power of GaAs substrates can be explained due to the lower absorption of light in the MIR region as well as the better thermal properties of the GaAs substrate compared to GaSb. Furthermore, measurement of recombination coefficients revealed that the shorter Shockley–Read–Hall recombination lifetime of the dislocation defects when grown on GaAs affects quantum efficiency only at low current injection. This finding opens the possibility of integrating cascaded SL-LEDs on a lower cost substrate (e.g., GaAs) than GaSb. Only 2 years later, the same group published another study exploiting the broad energy tunability of InAs/GaSb SLs on GaSb substrates to achieve an LED with eight different emission range levels in the range of 3–5 µm. [82] To do so, they connected eight regions of constant GaSb thickness (16 monolayers) and varying InAs thickness (6–9.7 monolayers) via tunnel crossings to reuse electrons in each emission region. Their measurements of the LED cooled to 77 K showed that at low current density $\left( {3.125\,{\text{A}}/{\text{cm}}^{2} } \right)$ the emission of each active region can be resolved as a monolithic emission region, but at high current $\left( {1000\,{\text{A}}/{\text{cm}}^{2} } \right)$ the individual peaks merge into a broad spectrum. In addition, at high current density, they saw an emissions peak at 3.7 µm and a resemblance of the LED spectrum to that of a blackbody spectrum at 1100 K.

**Fig. 6:**
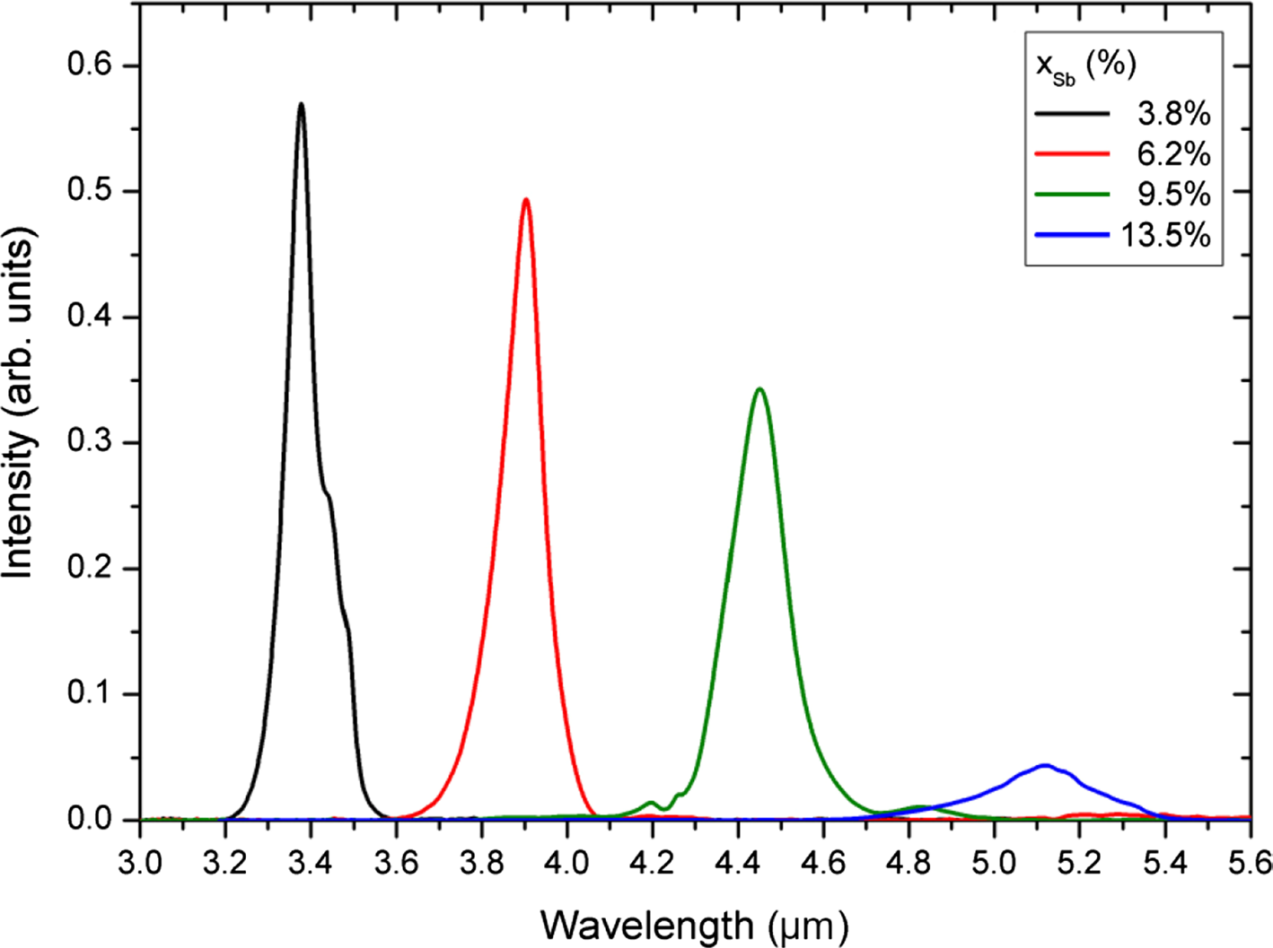
4 K photoluminescence spectra of InAs/InAs_1−x_Sb_x_ SLS structures with increasing Sb content. Copyright © 2017, IOP Publishing. Reprinted and edited with permission from [83].

Around the same time, Keen et al. [83] compared InAs/InAs_1-x_Sb_x_ superlattices and MQW structures LEDs for a Sb fraction of x = 3.8–13.5% at room temperature. They were able to show that both the 4 K photoluminescence spectra of the superlattice samples and the MQW samples showed peak shifts to longer wavelengths and a decrease in intensity as the antimony content was increased. The 4 K photoluminescence spectra of the superlattice samples are outlined in Fig. 6. Their simulations and measurements illustrate the effects by changing the structure by adjusting the Sb-content and layer thicknesses to maximize the radiative recombination rate. In addition, their photoluminescence studies demonstrated that the ground state transition from InAs to InAsSb existed in the SL structures up to room temperature. However, this was not seen in the MQW structures, which showed increasingly InAs-like behavior at temperatures above ~ 100 K. They were able to attribute this observation to a larger electron–hole overlap and stronger Coulomb attraction, which better confines the holes in the SL structure, allowing the transition to high temperatures. With their work, Keen and colleagues have thus shown a very good perspective for room temperature LED development using the SL-InAs/InAsSb structure.

In a more recent work, the research team around Kuze et al. [84] reported a high-intensity AlInSb midinfrared LEDs emitting at room temperature realized by adding InSb/AlInSb double buffer and electron/hole double barrier layers to improve crystalline quality and carrier confinement. Furthermore, by increasing the Al content (3.9–7.6%) in the Al_x_In_1-x_Sb buffer and active layer, they saw an emission peak shift to smaller wavelengths as well as an intensity increase, which is in contrast to Haigh et al. [76]. This difference can be explained by the roughening of the surface and TiO_2_ antireflection coating, which Haigh did not use. Based on their previous work, the same group published another paper in which they reduced Shockley–Read–Hall recombination in an Al_x_In_1-x_Sb (x = 5.7–12.1%) LED with central wavelength of 3.3 µm and thus achieved high luminosities [85]. By introducing dislocation filter layers of Al_y_In_1-y_Sb (y = 22–43%) between the active Al_x_In_1-x_Sb layers, they achieved dislocation bending at the Al_x_In_1-x_Sb/Al_y_In_1-y_Sb interfaces, reducing the dislocation density from $3.5 \cdot 10^{8} /{\text{cm}}^{2}$ to $1.1 10^{8} /{\text{cm}}^{2}$.

**Fig. 7:**
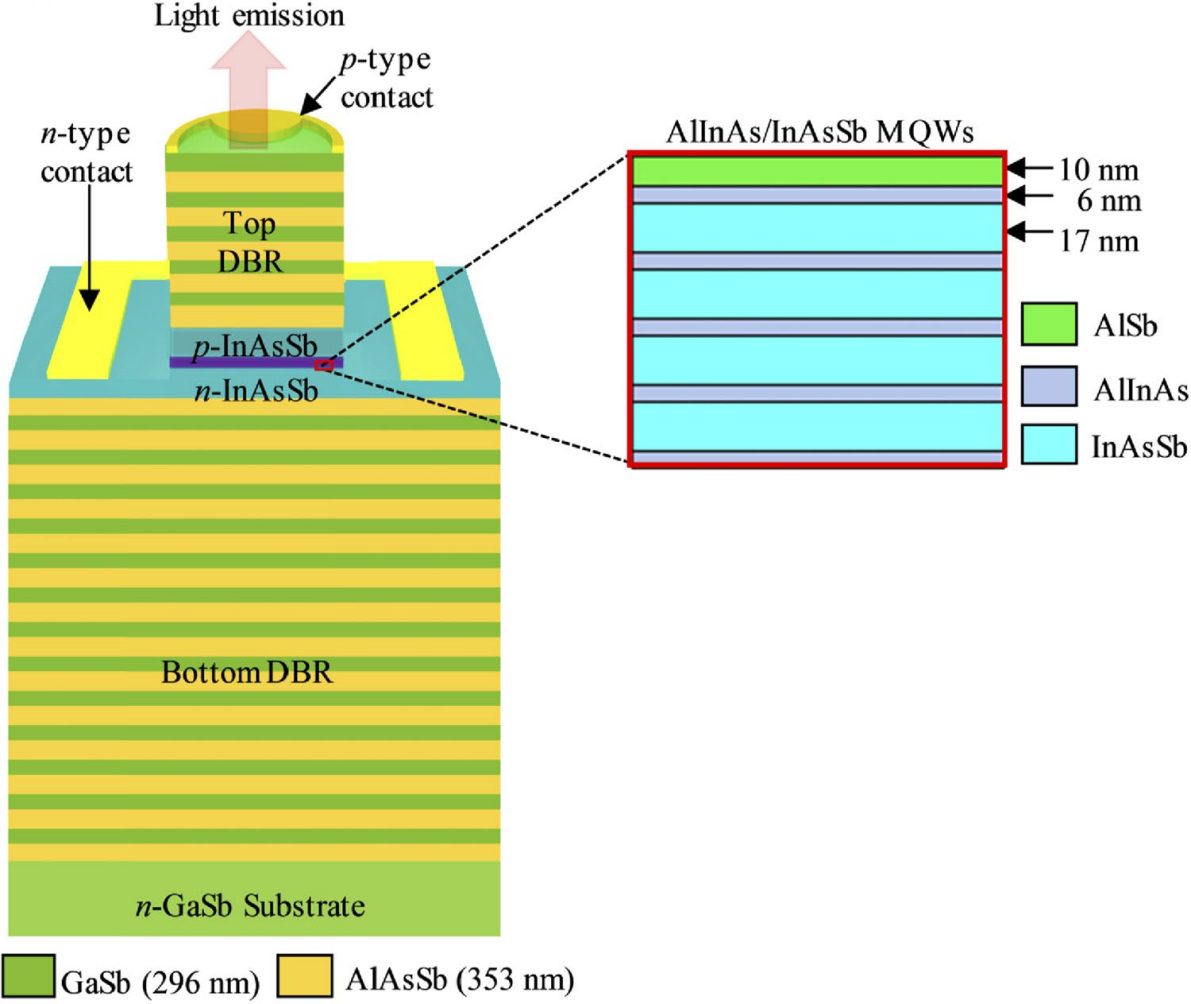
Schematic of a MQW resonant cavity LED structure showing the details of the AlInAs/InAsSb MQW in the active region of the p-i-n diode within the AlAsSb/GaSb DBRs, which form the microcavity. © 2020 Optica Publishing Group. Reprinted and edited with the permission from [87].

Most recently, efforts have been focused on mitigating the low output power with broadband emission spectra at room temperature for developing next-generation MIR-LEDs, and new approaches of structure modification to increase the power output have been devised. Al-Saymari's team presented two so-called resonant cavity LED (RCLED) systems [86, 87]. In these systems grown on GaSb substrate, the optical extraction efficiency limited by the large refractive index mismatch between the semiconductor and air has been addressed by placing the active structure between two DBRs. Figure 7 shows a schematic of the complete RCLED structure with top DBR/MQW/bottom DBR regions. Here, the first RCLED with a target wavelength of 4.2 µm consisted of lattice-matched AlAs_0.08_Sb_0.92_/GaSb DBR mirrors and a MQW InAs_0.90_Sb_0.10_ active region [86]. The second RCLED, on the other hand, had a target wavelength of 4.5 µm with AlAs_0.08_Sb_0.92_/GaSb DBR mirrors and MQW Al_0.12_In_0.88_As/InAs_0.85_Sb_0.15_ active region [87]. They could demonstrate for both RCLED systems that they have a significantly stronger electroluminescent as well as narrower emission spectrum compared to a reference MIR-LED without resonance cavity. However, they also saw a wavelength shift of the RCLEDs with increasing temperature.

Another recently explored method to increase the efficiency and performance of LEDs is to introduce stages into LED structure. For their system, Schaefer et al*.* [88] deposited three samples, each with nine stages (1 $\times$ 9 centered at the vibrational antinode, 3 $\times$ 3 positioned at the node, and 3 $\times$ 3 positioned at the vibrational antinode) in the GaSb/InAs active layer on a low-absorption substrate. They were able to show for their 640 $\times$ 640 µm sized system with a target wavelength of 3.5 µm that the electrical properties and decoupling efficiencies were improved when all active stages were centered within a single antinode of the optical field. The resulting optimization of the voltage efficiency as well as the lower optical losses led to a maximum radiant efficiency of 0.7% and a power output of maximal 5.1 mW at 0.6 A drive current.

Delli et al*.* [89] used a different approach to fabricate the MIR-LED heterostructures on a silicon substrate. For fabrication, they used a three-level buffer layer InAs/GaSB/Si (top to bottom). On this buffer layer, they deposited a five-period AlSb/InAs dislocation filter superlattices (DFSL), where each individual superlattice period again consisted of five alternating layers of AlSb and InAs. The individual superlattice periods were separated with a spacer layer of InAs. Finally, they deposited the InAs/InAsSb MQW structure on top of the DFSL. With their novel structure, they were able to show emission in the spectral range of 3.5–4.2 µm. Similar to what Kuze's team showed [85], Delli and colleagues also saw a strong filtering effect from filamentary dislocations at the InAs/GaSb interface.

Montealegre et al*.* [90] introduced even more stages in their system. They report a cascaded W-SL structure with an inner part consisting of 16 stages. Each of the stages in turn consists of an SL AlAsSb/InAs/GaInSb/InAs emission layer and an n/p GaSb/AlInAsSb tunnel junction. In addition, they thinned and roughened the emission side of the structure to improve light outcoupling, resulting in randomization of light direction and reduction of internal reflection and light outcoupling. With their 400 $\times$ 400 µm cascaded superlattice LED structure, they were able to demonstrate a power output of 6.8 mW at a wavelength of 4.2 µm in quasi-continuous wave mode (10 µs pulses) at room temperature.

## Selected applications

IR spectroscopy as a generic analytical technique may particularly benefit from the discussed advanced MIR light sources. By default, IR spectroscopy is based on broadband thermal infrared radiation. For target application scenarios, these may be replaced by non-coherent yet better tailorable IR light sources such previously described. Quantitative light-matter (i.e., molecule) interactions in IR spectroscopy are described using Lambert–Beer’s law:

3 $$A_{{\uplambda }} = { }\ln \left( {\frac{{I_{0} }}{{I_{1} }}} \right) = \varepsilon_{{\uplambda }} \cdot {\text{c}} \cdot {\text{d}}$$

with $A_{\lambda }$ the absorbance, *I*_0_ the intensity of the incident light of the source, $I_{1}$ the measured intensity, $\varepsilon_{\lambda }$ the absorption coefficient, *c* the concentration and *d* the length (i.e., absorption path length) of the irradiated sample.

The probably most common application of MIR spectroscopy in need for low-cost and efficient light sources is the detection of gaseous molecules in environmental analysis, process monitoring and biomedical diagnostics. In the infrared region, many prominent gases such as NH_3_ (2.1 μm), CH_4_ (2.35 μm and 3.3 μm), HCl (3.5 μm), N_2_O (3.9 μm and 4.5 μm), SO_2_ (4 μm), CO_2_ (4.2 μm), and CO (2.3 μm and 4.6 μm) have their molecule-specific vibrational, ro-vibrational, and rotational transitions just to name a few [91].

As previously described, currently many thermal MIR emitters as well as MIR-LEDs emit in a wavelength range of 4 ± 1 µm. Especially for MIR-LEDs, energy transitions of the semiconductor materials ≥ 6 µm remain a challenge, which is why to the best of our knowledge to date MIR-LEDs in the MIR range 6–20 µm have rarely been reported to date. Nevertheless, a sizeable number of gas measurements and sensors based on MEMS thermal emitters [92–96], plasmonic emitters [97, 98] and MIR-LEDs [2, 99–102] have already been demonstrated and developed based on currently accessible emission wavelengths. Among these devices, non-dispersive IR (NDIR) CO_2_ sensors are particularly common. CO_2_—with its emission peak ~ 4.2 µm—provides transitions ideally suited for the emission wavelengths of the presented light sources, and constitutes a prime target species for their application.

Two exemplary CO_2_ sensors reported in recent years are those of Vincent and Gardner [96] and Scholz et al. [103]. Both groups have described a year apart low-cost miniature CO_2_ sensor for mobile devices based on MEMS MIR emitter and MIR-LEDs, respectively.

Vincent and Gardner presented a MEMS thermal emitter-based NDIR sensor that was powered by current from a standard USB port and could measure CO_2_ concentrations in the range of 80 ppm—2.5% for a path length of 80 mm using a lock-in amplifier.

Scholz et al. on the other hand presented their progress for a MIR LED-based miniaturized NDIR CO_2_ sensor. By optimizing their waveguide/sample chamber combination, they were able to measure concentrations from 500 to 5000 ppm. Furthermore, they argued that by adjusting the duty cycle and sampling the energy consumption can be optimized for mobile device applications.

Besides CO_2_ detection, novel MIR light sources also play an important role in sensor development for monitoring the lower explosive limit of methane. Wittstock et al*.* [101] and Huang et al. [99] have presented two sensor designs based on MIR-LEDs with central emission wavelengths of 3.4 and 3.3 µm, respectively. In Wittstock's approach, they used a reflector–gas chamber combination, where the shape of the reflector consisted of two overlapping semi-ellipsoids. For optical path lengths of 23–36 mm, they had a detection limit of 2500 ppm methane even with the lowest path length. Huang and colleagues were even able to measure a methane detection limit of 17 ppb. However, they had used a significantly larger optical path length of 1 m in their sensor design using a hollow-core fiber.

Two other notable analytical application examples that used the novel narrowband MIR emitters in the LWIR were the NDIR scanning of organic liquids at 9 µm wavelength by Inoue et al*.* [63], and a MIR gas sensor for acetone and ammonia at 8.26 and 10.6 µm, respectively, by Xing et al. [97]. These groups used 2D PC and a plasmonic nanostructures, respectively, to achieve their emitter. Also, these studies impressively demonstrated qualitative and quantitative analyses using novel MIR emitters, and document their utility as a viable alternative to more commonly used laser sources.

Additional applications of the presented MIR emitters/absorbers worth mentioning here are beyond analytical scenarios and go beyond the scope of the present review, e.g., among others in military for stealth technology and thermovoltaics [3, 104, 105], and for power generation scenarios [44, 56, 106, 107].

## Conclusion and outlook

The mid-infrared spectral regime is the most information-rich part of the electromagnetic spectrum suitable for establishing sensing and diagnostic devices providing access to selective molecular absorption characteristics and multiple atmospheric transmission windows. Therefore, MIR devices—and the associated component technologies—are considered fundamental not only for chem/bio sensing, but also for imaging and communications applications. Key to both the fundamental and applied aspects of MIR technology are efficient radiation sources in the 2–20 µm wavelength range.

The present review discusses MIR light sources above and beyond devices resulting from the ‘laser revolution’ providing less intense yet tailorable emission characteristics with potentially low-cost per device, a tunable and confined emission range, and reduced power requirements during operation in miniaturized sensing and diagnostic systems. Table 1 gives an overview of the MIR light sources discussed in this review with their characteristics along with some prominent application examples.

**Table 1:** Overview of the discussed MIR light sources

Thermal emitter	Characteristics	Advantages	Disadvantages	Applications^a^
MEMS	Components structured and patterned using lithographic, etching and depositing methods in 1–100 µm range	Compatible with CMOS technology, low cost, low energy consumption	High driving temperature, need for heat resistant materials, low specific resistance, low oxidation rate	Optical gas sensing, air quality monitoring, NDIR gas detection
Plasmonic nanostructures	Periodic patterned (metallic) nano structures preventing ${\text{e}}^{ - }$ restoring ground state and instead are stimulated to oscillate at frequency of EM radiation	Tunable wavelength by changing surface periodicity, low cost	Oxidation at high temperature, T_max_ $\le$ 350 °C limiting output power	Environmental monitoring, solar thermo-photovoltaic systems
Thin film	Aperiodic multilayer structure of thin films; non-periodic number and thickness of alternating layers of conductor and isolator material	Continuously tunable emissivity through applied bias voltage or applied temperature; peak emissivity tunable through layer thickness	Complex manufacturing process due to alternating multilayer structure	Infrared stealth technology, temperature control of satellites, environmental observation
Photonic crystals	Periodic nanostructures that effect motion of phonons classified in 3D, 2D and 1D PC structures. PC influence EM radiation by allowed and forbidden energy bands through regularly repeating regions with high and low electric constant	High emissivity, tailorable wavelength and narrowband thermal emission, low energy consumption	High cost, complex size control on nanometer scale, limited in large area fabrication	Environmental sensing, radiative cooling, NDIR sensing
MIR LED	Broadband and incoherent light emission achieved by ${\text{e}}^{ - }$ hole recombination in type-II heterostructures or (multi) QW structures	Emission at room temperature, low electrical power consumption, easy implementation, low cost	Cooling needed in cw mode, mostly limited to 2–5 µm range, auger recombination strong limitation factor	NDIR gas sensing

^a^For several of the presented light source to date no specific target application has been reported that could be highlighted

Of particular emphasis are recent developments in the field of MIR-LED radiation sources, which not only provide advantageous properties, but may in future be augmented by appropriate organic materials (i.e., OLEDs) and quantum dots (i.e., QDs) leading to even more advanced technologies. While OLEDs have already been thoroughly researched emitting in the NIR [108–111], to date MIR-OLEDs have not been shown. In a similar fashion, NIR-QD emitters have readily been published [112–116], while the first MIR-QD system reported by Briggs et al*.* [117] in 2020 signals that MIR-QD emitters are on the horizon. Last but not least, expanding these concepts up to wavelengths around 20 µm is still pending, yet promises a highly active research field benefitting MIR chem/bio sensors and diagnostics in the years to come.
